# Trends in electrocardiographic abnormalities and risk of cardiovascular mortality in Lithuania, 1986–2015

**DOI:** 10.1186/s12872-019-1009-3

**Published:** 2019-01-30

**Authors:** Abdonas Tamosiunas, Janina Petkeviciene, Ricardas Radisauskas, Gailute Bernotiene, Dalia Luksiene, Mindaugas Kavaliauskas, Irena Milvidaitė, Dalia Virviciute

**Affiliations:** 1 Institute of Cardiology, Academy of Medicine, Lithuanian University of Health Sciences, Sukileliu av. 15, LT-50162 Kaunas, Lithuania; 2 Faculty of Public Health, Academy of Medicine, Lithuanian University of Health Sciences, Kaunas, Lithuania; 3 Faculty of Mathematics and Natural Sciences, Kaunas University of Technology, Kaunas, Lithuania

**Keywords:** Mortality, Electrocardiographic abnormalities, Risk factors, Trends, Cardiovascular disease, Middle-aged population

## Abstract

**Background:**

This study aimed to assess the trends in the prevalence of electrocardiographic (ECG) abnormalities from 1986 to 2015 and impact of ECG abnormalities on risk of death from cardiovascular diseases (CVD) in the Lithuanian population aged 40–64 years.

**Methods:**

Data from four surveys carried out in Kaunas city and five randomly selected municipalities of Lithuania were analysed. A resting ECG was recorded and CVD risk factors were measured in each survey. ECG abnormalities were evaluated using Minnesota Code (MC). Trends in age-standardized prevalence of ECG abnormalities were estimated for both sexes. Multivariate Cox proportional hazards models were used to estimate hazard ratios (HR) for coronary heart disease (CHD) and CVD mortality. Net reclassification index (NRI), integrated discrimination improvement and other indices were used for evaluation of improvement in the prediction of CVD and CHD mortality risk after addition of ECG abnormalities variable to Cox models.

**Results:**

From1986 to 2008, the decrease in the prevalence of Q-QS MC was observed in both genders. The prevalence of high R waves increased in men, while the prevalence of ST segment and T wave abnormalities as well as arrhythmias decreased in women. Ischemic changes and possible MI were associated with a 2.5-fold and 4.4-fold higher risk of death from CVD in men and 1.51-fold and 2.56-fold higher mortality risk from CVD in women as compared to individuals with marginal or no ECG abnormalities. The addition of ECG abnormalities to traditional CVD risk factors improved Cox regression models performance. According to NRI, 18.6% of men were correctly reclassified in CVD mortality prediction model and 25.2% of men - in CHD mortality prediction model.

**Conclusions:**

the decreasing trends in the prevalence of ischemia on ECG in women and increasing trends in the prevalence of left VH in men were observed. ECG abnormalities were associated with higher risk of CVD mortality. The addition of ECG abnormalities to the prediction models modestly improved the prediction of CVD mortality beyond traditional CVD risk factors. The use of ECG as routine screening to identify high risk individuals for more intensive preventive interventions warrants further research.

## Background

Cardiovascular diseases (CVD) are the main cause of death accounting for about one third of all deaths among both genders globally [1]. Deaths rates from CVD in Lithuania, a country of Eastern Europe, are significantly higher than in Northern, Southern and Western Europe countries [2]. In 2014, age-standardized mortality rate from CVD was 303.3 per 100,000 Lithuanian men aged 25–64 years as compared to 96.8 per 100,000 men in European Union countries. In women of the same age group, the difference in CVD mortality rate between Lithuania and EU was also high (75.0 and 32.1 per 100,000 women, respectively) [3].

The electrocardiography (ECG) is a routine, accessible, inexpensive, and non-invasive tool for diagnosis of CVD. Previous studies have reported that abnormalities on resting ECG have been independently associated with increased CVD risk [4–8]. However, the usefulness of ECG in screening of asymptomatic adults is still debatable because clinical implications of ECG abnormalities are unclear in low risk individuals [9]. Current evidence is insufficient to assess the benefits of ECG as screening tool in a population with high cardiovascular risk. Epidemiological studies carried out in Lithuanian adult population showed high prevalence of traditional CVD risk factors and ECG abnormalities [10, 11]. Considering this situation, incorporation of ECG abnormalities in CVD risk prediction might be useful in Lithuanian population. To date, few studies have analysed the improvement of CVD risk prediction adding ECG abnormalities to traditional CVD risk factors [12, 13].

Therefore, the aim of the present study was to estimate the trends in the prevalence of ECG abnormalities from 1986 to 2015 and the impact of ECG abnormalities on risk of death from CVD in the Lithuanian population aged 40–64 years.

## Methods

### Study sample

Four cross-sectional surveys of random samples representing the Lithuanian population aged 40–64 years were performed in 1986–1987, 1992–1993, 1999–2002, and 2006–2008. The surveys were carried out in Kaunas city and five municipalities of Lithuania from the northern, southern, eastern, western, and central regions of the country (Joniskis, Kaisiadorys, Kretinga, Kupiskis, and Varena). For each survey, an independent random sample, stratified by age and sex, was drawn from the Kaunas population register and from the lists of the individuals registered at the primary health care centres of municipalities. The selected individuals were invited to health examination sending them the invitation letters by mail. The response rates in the five cross-sectional surveys were 70.2, 69.6%, 58.6, 62.4, and 58.1% respectively. Participants with missing or inadequate ECG data (1.2%, similar numbers in all surveys) were excluded. Respondents with clinically diagnosed documented acute myocardial infarction (MI) were also excluded from analysis (the proportion of excluded respondents was 7.5% (8.5, 6.0, 7.1, 6.5, and 7.0% in the respective surveys). In total, data of 11,904 individuals (5427 men and 6477 women) were analysed.

All four studies were approved by the Lithuanian Regional Bioethics Committee. All participants signed informed consent form. Data of individual participants were not reported.

### Measurements

The measurements were taken in outpatient departments by the team consisting of trained doctors and nurses. In each survey, the measurements were performed using the same methodology. BP was measured twice from the right brachial artery with a standard mercury sphygmomanometer in the sitting position after 5 min of rest. Arterial hypertension (AH) was defined as systolic BP ≥140 mmHg and/or diastolic BP ≥90 mmHg, or BP < 140/90 mmHg taking antihypertensive medication for the last two weeks before medical examination.

Weight and height were measured with a stadiometer and a calibrated medical scale, without shoes and wearing light indoor clothing. Body mass index (BMI) was calculated as the ratio of weight (in kilograms) divided by the square of height (in meters) (kg/m^2^). A BMI within 18.5–24.99 kg/m^2^ was classified as normal weight, while a BMI within 25.0–29.99 kg/m^2^ was considered as overweight and participants with BMI ≥ 30.0 kg/m^2^ were classified as obese.

Fasting (at least 12 h) venous blood specimens were taken to determine lipid levels that were measured in blood serum by conventional enzymatic method in the certified laboratories. Dyslipidemias were defined as: high total cholesterol level – higher than or equal to 5.0 mmol/L; high low density lipoprotein (LDL) cholesterol level – higher than or equal to 3.0 mmol/L; low high density (HDL) cholesterol level – lower than 1.0 mmol/L for men and lower than 1.2 mmol/L for women, elevated triglycerides level – higher than or equal to 1.7 mmol/L.

Participants were classified as regular smokers (smoked at least one cigarette per day) and others.

A resting electrocardiogram was recorded in the 12 standard leads, with the calibration of 10 mm per 1 mV and paper speed of 25 mm per second. ECG records were read by 2 independent experienced coders (trained cardiologists) using the 1982 edition of the Minnesota Code (MC) [14]. Discrepancies between two coders were resolved by a senior coder (experienced cardiologist). The ECG findings were classified into 6 large groups [15]: 1) Q-QS waves – MC 1.1–1.3; 2) major ST segment and T wave changes – MC 4.1–4.3 or MC 5.1–5.3; 3) high R waves – MC 3.1 or MC 3.3; 4) left axis deviation – MC 2.1; 5) arrhythmias – MC 8.1–8.6 or MC 8.9 or MC 6.1–6.2 or MC 6.4 or MC 6.8; 6) blocks – MC 7.1 or MC 7.2 or MC 7.4. For further analysis, clinically meaningful combinations of ECG abnormalities were considered: 1) definite MI – MC 1.1 or MC (1.2 + 5.1 or 5.2); 2) possible MI - MC 1.3 + MC 5.1 or MC 5.2; or MC 1.2 alone; 3) left ventricular hypertrophy (VH) – MC 3.1 or 3.3 + any MC 4.1–4.3 or 5.1–5.3; 4) ischemia – MC 1.3 or MC 4.1–4.3 or MC 5.1–5.3; 5) marginal or no ECG abnormalities – all other codes. The five categories were hierarchically produced from the most to the least severe.

Follow up data of the individuals from Kaunas surveys were used for analysis of associations between ECG abnormalities and CHD or CVD mortality risk (*n* = 6090). Deaths were identified from the regional mortality register. Causes of death were coded by versions 9 and 10 of the International Classification of Diseases (ICD): CVD mortality included codes 390–458 of ICD-9 and I00-I99 of ICD-10; deaths from CHD included codes 410–414 of ICD-9 and I20-I25 of ICD-10. During period of 1986–2015, there were 1058 death cases from any cause, 503 deaths from CVD and 311 deaths from CHD.

### Statistical analysis

The statistical software package IBM SPSS Statistics 20 and the R statistical software were used for data analysis separately for men and women. We applied data weighting adjustment technique to match the age distribution of the Lithuanian population aged 40–64 years in 2008. The differences in age-adjusted means of variables between the surveys and between men and women were assessed using ANOVA analysis with Bonferroni multiple comparison test. A chi-squared test and z test with Bonferroni corrections were used for assessing the differences in categorical variables. *P* < 0.05 values were considered statistically significant.

Hazard ratios (HR) and 95% confidence intervals (CI) were estimated by the multivariate Cox proportional hazards regression for CVD and CHD mortality. First of all, HRs were calculated for five categories of clinically meaningful combinations of ECG abnormalities with adjustment for study number and CVD risk factors, such as age, AH, smoking, HDL cholesterol, LDL cholesterol, triglycerides, and BMI. Later, the predictive ability of Cox regression Model 1, which included only above mentioned CVD risk factors, was compared with predictive ability of the Model 2 extended by ECG abnormalities. ECG abnormalities were categorized into two groups – 1) absent or marginal ECG abnormalities and 2) any of clinically meaningful ECG abnormality. The additional predictive value of the models was determined using log-likelihood ratio (likelihood ratio test - LRT). Lower *P* values indicated larger difference between predictive quality of the models. The incremental prognostic impact of the new predictor (ECG abnormality) was estimated with Harrel’s C statistic, integrated discrimination improvement (IDI), and the net reclassification improvement index (NRI) [16, 17]. Harrel’s C statistic is an established measure of model discrimination for binary outcomes and is assessed by calculating the area under the receiver-operating characteristic curve. IDI is a relatively independent of risk thresholds and categories measure of the ability of the extended model to improve average sensitivity without compromising average specificity. NRI was calculated to determine the extent to which incorporation of additional variable into the prediction models improve the reclassification of individuals. IDI and NRI were calculated at 10 years of follow-up.

## Results

Age-standardized prevalence of ECG abnormalities among Lithuanian men and women aged 40–64 years is presented in Table 1. The prevalence of Q-QS codes significantly decreased from 4.1% in 1986–1987 to 2.1% in 2006–2008 in men and from 3.2 to 2.1% in women. In men, the prevalence of left axis deviation and high R waves showed a significant increase between the first and last survey, while no significant trends in those abnormalities were found in women. A significant decrease in the prevalence of ST segment and T wave abnormalities and arrhythmias in women was observed from 1986 to 1987 to 2006–2008.

**Table 1:** Age-standardized prevalence of ECG abnormalities in Lithuanian population aged 40–64 years

Prevalence of ECG abnormalities	Study years				*P* from chi squared
	1986–1987	1992–1993	1999–2002	2006–2008	
Men (*n*)	**1496**	**926**	**1119**	**1886**	
Q - QS codes, %	4.1	3.8^*^	2.5^*^	2.1^a^	**0.002**
Left axis deviation, %	4.3^*^	5.5^*^	3.3	5.2^*^	**0.048**
ST-T codes, %	6.3^*^	3.8^a, *^	5.0^*^	5.1	0.057
High R waves, %	6.3^*^	5.8^*^	9.0^a, b, *^	9.5^a, b, *^	**< 0.001**
Arrhythmia, %	5.3	5.9	4.7^*^	4.4^*^	0.295
Blocks, %	1.4	1.4^*^	1.8^*^	1.8^*^	0.729
Definite myocardial infarction, %	1.2^*^	1.0^*^	0.8^*^	0.5^*^	**< 0.001**
Possible myocardial infarction, %	1.2	1.1	0.7	0.7^*^	
Left ventricular hypertrophy, %	3.1	4.0^*^	6.3 ^a, *^	5.8 ^a, *^	
Ischemia, %	5.9^*^	4.0^*^	4.0^*^	4.7	
Marginal abnormalities or absent, %	88.6*	89.8	88.1	88.3*	
Women (*n*)	**1609**	**1114**	**1415**	**2339**	
Q - QS codes, %	3.2	1.8	1.1^a^	2.1	**< 0.001**
Left axis deviation, %	2.8	2.8	2.2	2.7	0.696
ST-T codes, %	12.9	7.7^a^	9.1^a^	5.9^a, c^	**< 0.001**
High R waves, %	4.2	2.7	3.7	3.5	0.233
Arrhythmia, %	4.8	4.2	2.6^a^	3.1^a^	**0.004**
Blocks, %	0.7	0.5	0.6	0.8	0.869
Definite myocardial infarction, %	0.4	0.0	0.2	0.2	**< 0.001**
Possible myocardial infarction, %	1.4	0.6	0.4^a^	1.4^c^	
Left ventricular hypertrophy, %	2.6	1.7	2.1	2.2	
Ischemia, %	12.3	8.2^a^	8.4^a^	5.9^a, c^	
Marginal abnormalities or absent, %	83.2	89.5^a^	88.9^a^	90.3^a^	

^a^*p* < 0.05 compared to 1986–1987, Bonferroni test. ^b^*p* < 0.05 compared to 1992–1993, Bonferroni test. ^c^*p* < 0.05 compared to 1999–2002, Bonferroni test. *p < 0.05 compared men to women

ECG: electrocardiogram

Bold typeface of *P* values indicates significance

In the majority of the surveys, the prevalence of ST segment and T wave abnormalities was significantly higher in women than men, while the prevalence of other ECG abnormalities was higher in men compared to women (Table 1).

Analysis of clinically meaningful ECG abnormalities showed that the prevalence of left VH in men increased from 3.1% in 1986–1987 to 5.8% in 2006–2008, whereas the prevalence of ischemia in women decreased from 12.3 to 5.9% during the same period.

Age-standardized mean levels and the prevalence of main risk factors among men and women were analysed using the pooled data from all surveys (Tables 2 and 3). The men with definite MI were older than men with marginal or absent ECG abnormalities. They had higher mean total and LDL cholesterol levels and lower level of systolic BP as compared to men with left VH. In men with ischemia, mean levels of systolic and diastolic BP, BMI and triglycerides were higher than in men with marginal or absent ECG abnormalities.

**Table 2:** Age-standardized mean values and prevalence of risk factors in men by ECG abnormality group

ECG abnormalities						
Mean values and prevalence	Definite MI	Possible MI	Left VH	Ischemia	Marginal or absent	*P* from Anova
	*n* = 45	*n* = 51	*n* = 263	*n* = 258	*n* = 4810	
Age, years, mean (SD)	54.7 (6.49)	53.9 (6.25)	51.3^a^ (7.44)	54.2^c^ (6.88)	51.1^a,b,d^ (6.87)	**< 0.001**
Systolic BP, mmHg, mean (SD)	142.9 (25.7)	152.1 (32.)	153.2^a^(25.4)	154.7 ^a^ (24.5)	142.7^b,c,d^ (20.9)	**< 0.001**
Diastolic BP, mmHg, mean (SD)	91.3 (13.0)	94.7 (21.4)	97.4 (13.0)	96.6 (14.0)	91.3^c,d^ (12.4)	**< 0.001**
BMI, kg/m^2^, mean (SD)	27.6 (4.82)	29.0 (4.43)	27.2 (4.04)	29.2 ^c^ (4.78)	27.6^d^ (4.56)	**< 0.001**
Total cholesterol, mmol/L, mean (SD)	6.29 (1.21)	6.12 (1.31)	5.73^a^ (1.16)	6.03 (1.23)	5.88 (1.20)	**0.007**
LDL cholesterol, mmol/L, mean (SD)	4.27 (1.16)	4.00 (1.08)	3.63^a^ (1.13)	3.92 (1.11)	3.81 (1.17)	**0.006**
HDL cholesterol, mmol/L, mean (SD)	1.26 (0.27)	1.17 (0.33)	1.39^b^ (0.39)	1.27 ^c^ (0.45)	1.35 (0.43)	**0.001**
Triglycerides, mmol/L, mean, (SD)	1.37 (0.58)	2.04^a^ (1.73)	1.63 (0.93)	1.83 (1.12)	1.62^d^ (1.02)	**0.001**
						*P* from χ^2^
Arterial hypertension, %	63.3	82.4	80.5	83.3^a^	64.7^c,d^	**< 0.001**
Regular smoking, %	35.6	25.5	37.8	41.1	39.7	0.274
Total cholesterol > 5.0 mmol/L, %	86.0	87.2	71.5	79.3	76.9	**0.045**
LDL cholesterol > 3.0 mmol/L, %	88.6	86.0	66.9	82.7^c^	75.4^c^	**< 0.001**
HDL cholesterol < 1.0 mmol/L, %	16.7	32.6	11.1^b^	29.1^c^	19.5^c, d^	**< 0.001**
Triglycerides > 1.7 mmol/L, %	26.2	48.9	37.4	44.6	34.1^d^	**0.002**
Normal weight, BMI < 25.0 kg/m^2^, %	29.5	13.7	29.0	19.4	29.2^d^	**< 0.001**
Overweight, BMI 25.0–29.9 kg/m^2^,%	50.0	47.1	48.5	42.2	44.7	
Obesity, BMI > 30.0 kg/m^2^, %	20.5	39.2	22.5	38.4^c^	26.1^d^	

^a^*p* < 0.05 compared to definite MI group, Bonferroni test. ^b^*p* < 0.05 compared to possible MI group, Bonferroni test. ^c^*p* < 0.05 compared to left VH group, Bonferroni test, ^d^*p* < 0.05 compared to left VH group, Bonferroni test. *BMI* body mass index, *BP* blood pressure, *ECG* electrocardiogram, *HDL* high-density lipoproteins, *MI* myocardial infarction, *LDL* low-density lipoproteins, *SD* standard deviation, *VH* ventricular hypertrophy

Bold typeface of *P* values indicates significance

**Table 3:** Age-standardized mean values and prevalence of risk factors in women by ECG abnormality group

ECG abnormalities						
Mean values and prevalence	Definite MI	Possible MI	Left VH	Ischemia	Marginal or absent	*P* from Anova
	*n* = 14	*n* = 69	*n* = 584	*n* = 105	*n* = 5705	
Age, years, mean (SD)	53.0 (6.40)	51.0 (7.41)	54.3^b^ (6.66)	53.9^b^ (6.62)	50.9^c,d^ (6.91)	**< 0.001**
Systolic BP, mmHg, mean (SD)	168.9 (38.9)	150.3 (28.2.)	162.5^b^ (27.1)	146.3^a,c^ (25.8)	139.1^a,b,c,d^ (22.8)	**< 0.001**
Diastolic BP, mmHg, mean (SD)	98.8 (19.0)	91.4 (14.4)	97.0^b^ (13.7)	90.2^c^ (13.0)	87.5^a,c,d^ (12.1)	**< 0.001**
BMI, kg/m^2^, mean (SD)	32.3 (6.79)	30.1 (5.14)	31.3 (6.28)	30.2 (5.71)	29.2^c,d^ (5.63)	**< 0.001**
Total cholesterol, mmol/L, mean (SD)	5.68 (1.08)	5.78 (1.39)	6.14 (1.21)	6.40^b^ (1.47)	5.99^d^ (1.27)	**< 0.001**
LDL cholesterol, mmol/L, mean (SD)	3.12 (0.99)	3.56 (1.29)	3.95 (1.19)	4.22^a,b^ (1.39)	3.83^d^ (1.21)	**< 0.001**
HDL cholesterol, mmol/L, mean (SD)	1.66 (1.02)	1.41 (0.39)	1.51 (0.41)	1.47 (0.41)	1.51 (0.41)	0.072
Triglycerides, mmol/L, mean, (SD)	1.49 (0.77)	1.53 (1.00)	1.54 (0.74)	1.60 (0.95)	1.45^d^ (0.77)	**0.001**
						*P* from χ^2^
Arterial hypertension, %	85.7	68.2	86.2^b^	65.0^c^	55.4^c,d^	**< 0.001**
Regular smoking, %	21.4	8.7	7.0	5.7	9.1	**0.030**
Total cholesterol > 5.0 mmol/L, %	76.9	72.1	85.9	85.3	78.6^d^	**0.001**
LDL cholesterol > 3.0 mmol/L, %	54.5	70.0	77.3	82.9	74.6^d^	**0.001**
HDL cholesterol < 1.0 mmol/L, %	41.7	32.3	21.5	25.2	22.3	0.072
Triglycerides > 1.7 mmol/L, %	25.0	24.2	31.3	34.5	28.5	0.065
Normal weight, BMI < 25.0 kg/m^2^, %	7.7	17.1	15.6	19.7	24.2	**< 0.001**
Overweight, BMI 25.0–29.9 kg/m^2^,%	23.1	30.0	30.0	31.1	36.3	
Obesity, BMI > 30.0 kg/m^2^, %	69.2	52.9	52.9	53.9	39.5^c,d^	

^a^*p* < 0.05 compared to definite MI group, Bonferroni test. ^b^*p* < 0.05 compared to possible MI group, Bonferroni test. ^c^*p* < 0.05 compared to left VH group, Bonferroni test, ^d^*p* < 0.05 compared to left VH group, Bonferroni test. *BMI* body mass index, *BP* blood pressure, *ECG* electrocardiogram, *HDL* high-density lipoproteins, *MI* – myocardial infarction, *LDL* low-density lipoproteins, *VH* ventricular hypertrophy

Bold typeface of *P* values indicates significance

The women with definite MI had higher means of systolic and diastolic BP as compared to women with ischemia and marginal or no ECG abnormalities (Table 3). The highest level of LDL cholesterol was observed in women with ischemia. Moreover, those women had higher BMI, total cholesterol and triglycerides than women having marginal or no ECG abnormalities.

Cox regression analysis was used to calculate the independent contribution of clinically meaningful ECG abnormalities to the risk of dying from CVD and CHD when other conventional risk factors had been accounted for (Table 4). Follow up period started from the health examination date and finished on 31st of December, 2015. The mean duration of follow-up was 12.8 + 7.79 years (12.5 + 7.82 years in men and 13.1 + 7.75 years in women). In men, ischemic changes on ECG and possible MI were associated with a 2.5-fold and 4.4-fold higher risk of death from CVD as compared to men with marginal or absent of ECG abnormalities (reference group). Risk of dying from CVD was 1.51-fold higher in women with ischemia and 2.56-fold higher in women with possible MI than in reference group. A similar impact of clinically meaningful ECG abnormalities on CHD mortality was observed in men but not in women (Table 4).

**Table 4:** Adjusted^a^ hazard ratios of CVD and CHD mortality by group of clinically meaningful electrocardiographic abnormalities and sex

	CVD mortality				CHD mortality			
ECG abnormality	Men *n* = 281		Women *n* = 167		Men *n* = 172		Women *n* = 96	
	HR (95% CI)	*p* value	HR (95% CI)	*p* value	HR (95% CI)	*p* value	HR (95% CI)	*p* value
Marginal abnormalities or absent	1.0	–	1.0	–	1.0	–	1.0	–
Ischemia	**2.50 (1.67–3.74)**	**< 0.001**	**1.51 (1.00–2.27)**	**0.048**	**2.00 (1.14–3.51)**	**0.016**	1.67 (0.99–2.82)	0.053
Left VH	1.65 (0.94–2.91)	0.082	1.19 (0.52–2.72)	0.686	1.58 (0.77–3.25)	0.211	1.09 (0.34–3.50)	0.885
Possible MI	**4.40 (2.30–8.42)**	**< 0.001**	**2.56 (1.03–6.35)**	**0.042**	**4.83 (2.21–10.55)**	**< 0.001**	3.04 (0.94–9.86)	0.064
	**(2.30–8.42)**		**(1.03–6.35)**		**(2.21–10.55)**		(0.94–9.86)	
Definite MI	1.94 (0.48–7.91)	0.355	3.75 (0.50–27.93)	0.197	2.83 (0.68–11.68)	0.151	6.81 (0.88–52.76)	0.066

^a^ - adjusted for age, education, arterial hypertension, high density lipoprotein cholesterol, low density lipoprotein cholesterol, body mass index, smoking, and study number. *CI* confidence interval, *CHD* coronary heart disease, *CVD* cardiovascular diseases, *ECG* electrocardiogram, *HR* hazard ratio, *MI* myocardial infarction, *VH* ventricular hypertrophy. Bold typeface indicates significance

For comparison of predictive ability of Cox regression models, the dichotomized variable (any clinically meaningful ECG abnormality and absent of abnormality) was used. Risk of CVD and CHD mortality was significantly higher in men and women having any clinically meaningful ECG abnormality (Table 5). The addition of ECG abnormalities had some effect on Cox regression models performance. According to the LRT tests, the extension of the Model 1 by mentioned variable significantly improved the performance of model in both genders. A slight increase in Harrell’s C-statistic was observed, once the variable of ECG abnormalities was added to other risk factors in the models for CVD and CHD mortality. In men, the values of IDI were 1.3% (*p* = 0.007) for CVD mortality and 1.0% (*p* < 0.001) for CHD mortality, which suggests a modest improvement in discrimination of the Cox regression models when ECG abnormalities were added to the models. In women, the respective values of IDI were lower than in men. Addition of ECG abnormalities to the model adjusted for traditional CVD risk factors resulted in reclassification of 18.6% of men in CVD mortality prediction model and 25.2% of men in CHD mortality prediction model. No net reclassification improvement was observed in women.

**Table 5:** Comparison of models for prediction of CVD and CHD mortality without and with addition of electrocardiographic abnormalities

ECG	CVD mortality				CHD mortality			
abnormalities	Men *n* = 281		Women *n* = 167		Men *n* = 172		Women *n* = 96	
	Model 1	Model 2	Model 1	Model 2	Model 1	Model 2	Model 1	Model 2
		HR (95% CI)		HR (95% CI)		HR (95% CI)		HR (95% CI)
No	–	1	–	1	–	1	–	1
Yes	–	**2.34 (1.72–3.19)**	–	**1.56 (1.09–2.24)**	–	**2.15 (1.44–3.22)**	–	**1.71 (1.07–2.73)**
Harrell‘s C	0.704	0.730	0.803	0.814	0.715	0.745	0.829	0.842
LRT (*p*-value)	–	**24.62 (< 0.001)**	–	**5.48 (0.019)**	–	**12.02 (< 0.001**)	–	**4.79 (0.029)**
IDI (*p*-value)	–	**0.013 (0.007)**	–	**0.004 (0.040)**	–	**0.010 (< 0.001)**	–	0.004 (0.173)
NRI (*p*-value)	–	**0.186 (0.007)**	–	0.186 (0.153)	–	**0.252 (0.027)**	–	0.212 (0.206)

Model 1 – factors included into model: age, education, arterial hypertension, high density lipoprotein cholesterol, low density lipoprotein cholesterol, triglycerides, body mass index, smoking; Model 2 – variables of Model 1 plus ECG abnormalities

*CHD* coronary heart disease, *CI* confidence interval, *CVD* cardiovascular diseases, *HR* hazard ratio, *IDI* integrated discrimination improvement, *LRT* likelihood ratio test, *NRI* net reclassification index

Bold typeface indicates significance

## Discussion

This study describes the trends in the prevalence of ECG abnormalities and the prognostic impact of these abnormalities on risk of death from CHD and CVD in Lithuania over a period of more than two decades. To our knowledge, our study is the first to describe trends in the prevalence and prognostic impact of ECG abnormalities coded by MC not only in Lithuania but also in all Baltic countries.

Over the study period, we observed a significant increase in the prevalence of left VH in male population. These findings might be explained by the data of our previous studies carried out during the same period showing the increasing trends in the prevalence of AH among middle-aged Lithuanian men [11]. Other investigators demonstrated that effective antihypertensive treatment might result in regression of left VH [18, 19]. In Lithuania, however, the proportion of controlled AH still remains rather low, especially among men, despite some improvement in AH management [20].

During 20 years period, the prevalence of ischemia among women decreased. The observed positive trends might be associated with a significant decrease in the prevalence of dyslipidemias and obesity in Lithuanian women between 1986 and 2006 [11]. The positive changes in lipid levels could be related to the changes in nutrition habits of Lithuanians that were more pronounced in women. Our previous study demonstrated the increase in the use of vegetable oil for cooking and replacement of butter spread with margarine, also the decrease in consumption of high fat milk [21]. Moreover, the National CVD Prevention Programme for high risk individuals was introduced in Lithuania in order to improve the control of CVD risk factors [22]. The reduced prevalence of ischemia on ECG of women was associated with decreasing trends in CVD mortality among Lithuanian women between 1985 and 2013 [11]. In men, the reduction of CVD mortality was observed only from 2008. These changes could be attributed to a combination of long-term increase in income, other socio-economic changes, improvements in health care and dietary changes.

In our study, the prevalence of ECG abnormalities, with exception of ST-T codes, was significantly higher in men than in women. Similar gender differences in the prevalence of major and minor ECG abnormalities were found in the adult population of Reykjavik and in the Hispanic Community Health Study [6, 23]. However, in the Chicago Heart Association Detection Project the prevalence rates of major ECG abnormalities were higher in women than in men, while minor ECG abnormalities were more common in men compared to women [24].

We found lower mean levels of systolic and diastolic BP, some lipids and BMI in men and women with marginal or absent ECG abnormalities as compared to individuals having ECG abnormalities. Our data is in line with the findings from other studies where the prevalence and mean levels of most CVD risk factors were higher in individuals with major and minor ECG abnormalities as compared to those without such abnormalities [25–28]. On the other hand, the prevalence of ECG abnormalities has been found higher in individuals with CVD risk factors [29].

After adjustment for the main CVD risk factors, male participants of our study with ischemia and possible MI based on ECG abnormalities had a significantly higher risk of CVD and CHD mortality as compared to men with marginal or absent ECG abnormalities. In women, the same ECG abnormalities were associated only with higher risk of CVD mortality; moreover, HRs were lower than in men. Our results are similar to those obtained in other population studies that analysed gender differences in prognostic values of ECG abnormalities and showed lower CVD mortality risk in women than in men [24, 30]. However, other authors did not find any gender differences in predicting CVD mortality risk for ECG abnormalities [31–34].

Pathological Q waves in the absence of documented MI are important cardiac abnormalities detected through ECG screening. Those patients do not receive medical treatments that could prevent adverse outcomes, including recurrent MI or CVD death [35]. Cohort studies demonstrated that Q/QS wave abnormalities (silent MI) were associated with increased risk of CHD and all-cause mortality [36–38]. Prognostic values of those ECG abnormalities have been shown to be similar to clinically documented MI.

Nonspecific ST segment and T wave abnormalities were related to CVD and CHD mortality in asymptomatic older and middle-aged persons [32, 39–41]. It was suggested that those ECG abnormalities might indicate subclinical CHD.

Several studies reported that ECG left VH was a predictor of multiple CVD outcomes (sudden cardiac death, MI, congestive heart failure and stroke) [25, 42]. According to results of the LIFE study, regression of ECG left VH during antihypertensive treatment was associated with better CVD outcomes [18]. Contrary to those findings, we did not estimate an association between left VH and mortality from CVD and CHD. Similar results were found in the study conducted in three European countries [15]. Possible explanation could be an adjustment of mortality HRs for AH which is strongly related with left VH.

Our results demonstrated that resting ECG abnormalities significantly improved the prediction of CVD and CHD mortality beyond traditional cardiovascular risk factors. In men, the addition of ECG abnormalities resulted in reclassification of 18.6 and 25.2% of participants when risk of dying from CVD and CHD was assessed. Only few studies analysed the changes in the predictive ability of Cox models after the addition of resting ECG abnormalities [12, 13]. The results of Health, Aging, and Body Composition Study (Health ABC Study) showed that 13.6% participants with minor and major ECG abnormalities were correctly reclassified when ECG abnormalities were added [39]. However, in studies carried out in Netherlands and Norway, inclusion of ECG abnormalities did not improve models performance [13, 43].

Some limitations should be considered when interpreting our findings. Only a single ECG tracing was recorded for each participant during the baseline examination. It is recognized that single ECG record, same as other single biologic measurements, could lead to underestimation of the impact of examined factors on risk due to misclassification [44]. The low prevalence of some ECG abnormalities could be another limitation of our study. A relatively small number of deaths within ECG categories may limit the power to demonstrate the association between ECG abnormalities and mortality risk. Moreover, we have no information about changes in participants’ health behaviours, biologic CVD risk factors, and ECG during observation period. These changes might have impact on risk of mortality from CVD. Finally, the response rates lower than 70% might have resulted in selection bias and potential underestimation of the prevalence of ECG abnormalities, if non-response was associated with a more adverse risk profile. However, it is unlikely that the strength of this association has changed substantially between the surveys. This assumption together with the relatively similar response rates over time minimizes the potential impact of non-response on our estimates of the trends in the prevalence of ECG abnormalities.

The strength of our study includes the large number of participants from middle-aged Lithuanian population and long-term follow up. To our knowledge, this is the first report on the trends in ECG abnormalities and impact of these abnormalities on risk of death from CHD and CVD in Lithuania and other Baltic countries. The standardized ECG procedures and carefully documented registration of death events during follow up were used. Multiple lifestyle and biological CVD risk factors were assessed using uniform collection of data. This allowed to make adjustments for a large number of potential confounding variables in the Cox models.

## Conclusions

In 1986–2008, the decreasing trends in the prevalence of ischemia on ECG in women and increasing trends in the prevalence of left VH in men were observed. ECG abnormalities were associated with higher risk of CVD mortality and modestly improved the prediction of CVD mortality beyond traditional CVD risk factors. Given the wide availability, low cost and safety, the ECG might be used to improve CVD risk prediction and to identify the individuals for more intensive preventive interventions. Further studies are needed to ascertain whether ECG should be incorporated in routine screening.

## Abbreviations

AH: Arterial hypertension
BMI: Body mass index
BP: Blood pressure
CHD: Coronary heart disease
CI: Confidence interval
CVD: Cardiovascular diseases
ECG: Electrocardiographic
HDL: High density lipoproteins
HR: Hazard ratio
ICD: International classification of diseases
IDI: Integrated discrimination improvement
LDL: Low density lipoproteins
LRT: Log-likelihood ratio
MC: Minnesota code
MI: Myocardial infarction
NRI: Net reclassification improvement index
SD: Standard deviation
VH: Ventricular hypertrophy
